# Developing a Tool to Measure Person-Centered Care in Service Planning

**DOI:** 10.3389/fpsyt.2021.681597

**Published:** 2021-08-02

**Authors:** Victoria Stanhope, Daniel Baslock, Janis Tondora, Lauren Jessell, Abigail M. Ross, Steven C. Marcus

**Affiliations:** ^1^Silver School of Social Work, New York University, New York, NY, United States; ^2^Program of Recovery and Community Health, Yale School of Medicine, Yale University, New Haven, CT, United States; ^3^Graduate School of Service, Fordham University, New York, NY, United States; ^4^School of Social Policy and Practice, University of Pennsylvania, Philadelphia, PA, United States

**Keywords:** person-centered care, person-centered care planning, community mental health, mental health services, measurement, service planning

## Abstract

**Background:** Delivering person-centered care is a key component of health care reform. Despite widespread endorsement, medical and behavioral health settings struggle to specify and measure person-centered care objectively. This study presents the validity and reliability of the Person-Centered Care Planning Assessment Measure (PCCP-AM), an objective measure of the extent to which service planning is person-centered.

**Methods:** Based upon the recovery-oriented practice of person-centered care planning, the 10-item PCCP-AM tool rates service plans on the inclusion of service user strengths, personal life goals, natural supports, self-directed actions and the promotion of community integration. As part of a large randomized controlled trial of person-centered care planning, service plans completed by community mental health clinic providers were rated using the PCCP-AM. Reliability was tested by calculating inter-rater reliability across 168 plans and internal consistency across 798 plans. To test concurrent validity, PCCP-AM scores for 84 plans were compared to expert rater scores on a separate instrument.

**Results:** Interrater reliability for each of the 10 PCCP-AM items as measured by Kendall's *W* ranged from *W* = 0.77 to *W* = 0.89 and percent of scores within ± 1 point of each other ranged from 85.7 to 100%. Overall internal consistency as measured by Cronbach's alpha across 798 plans was α = 0.72. Concurrent validity as measured by Kendall's *W* ranged from *W* = 0.55 to *W* = 0.74 and percent of item scores within ± 1 point of expert rater scores ranged from 73.8 to 86.8%.

**Conclusions:** Findings demonstrated that the 10-item PCCP-AM was a valid and reliable objective measure of person-centered care. Using the service plan as an indicator of multiple domains of person-centered care, the measure provides a valuable tool to inform clinical supervision and quality improvement across programs. More psychometric testing is needed to strengthen the measure for research purposes.

## Introduction

There has been a growing recognition that patient-centered care is integral to health care reform efforts. The Institute of Medicine (1) has defined patient-centered care as “providing care that is respectful of and responsive to individual patient preferences, needs and values” (p.3). Also referred to as “person-centered” care in behavioral health settings to convey a more active role for the individual receiving care, the approach embraces a holistic understanding of wellness rather than reducing care to treating isolated symptoms (2). While receiving widespread endorsement both in medical and behavioral health care settings, how this individualized and contextual approach to health care translates to specific clinical practices has been less clear. As health care systems are faced with increasing demands to demonstrate and document person-centered care, there is a need to specify and objectively measure this approach (3). This article describes the development and validation of the Person-Centered Care Assessment Measure, an objective measure of person-centered care based upon documentation within mental health settings.

Person-centered care (PCC) is one of the key aims for health care reform (1). Essentially a values-based approach, person-centered care challenges the disease-centered approach of the medical model and empowers individuals to make decisions about their treatment. In the United States, the Affordable Care Act has provided incentives for new health care models to deliver person-centered care and globally, the World Health Organization has articulated a vision for integrated people centered health services (4). Initiatives in the United Kingdom include the Health and Social Care Act 2012, which requires NHS England to involve people in their care and similar policies exist in Scotland, Northern Ireland and Wales (5). In Australia, patient-centered approaches are supported by the Australian Charter of Healthcare Rights and the National Quality and Safety Health Services Standards (6).

There is a growing evidence base demonstrating that person-centered approaches can improve an individual's self-management and treatment engagement, as well as their overall satisfaction and the perceived quality of care (7–9). Within mental health settings, empowering people to make decisions about their care has been shown to increase engagement in therapeutic (10) and psychiatric treatment (11), reduce symptom severity (12), increase medication adherence (13) and increase client reports of well-being (14). However, as some systematic reviews have concluded, positive outcomes are not consistent across studies, despite efforts to conceptualize and operationalize person-centeredness in mental health (15).

Part of the challenge lies with how intuitive and self-evident the idea of person-centeredness can be for healthcare providers. Many providers feel that they are “already doing it” and so are resistant to efforts to make their practice more person-centered (16). In turn, when providers are asked to self-report their person-centeredness, they tend to endorse high levels of PCC even when objective indicators suggest otherwise (17) undermining efforts to accurately evaluate PCC. Currently, the large majority of person-centered care measures rely on self-report creating a need for objective measures.

Person-centered care has been conceptualized as one aspect of service quality (18). When considering how to capture the implementation of an evidence-based practice, some researchers have conceptualized service quality as a service outcome, which is predicted by implementation outcomes such as adoption, penetration, fidelity and sustainability (19). Whereas others have posited that service quality is an aspect of fidelity, referring to the extent to which a provider adheres to techniques and the theoretical ideal of an intervention (20, 21). A common critique of fidelity measures is that they have focused more on structure than process, despite the fact that the less tangible elements of a program maybe their most essential aspects (22). While our understanding of how service quality fits into implementation frameworks are unresolved, there remains an urgent need for effective measures of the more nuanced but highly valued process aspects of service delivery such as person-centered care.

While PCC is more emphasized in certain practices than others, it is increasingly an aspiration for service delivery generally and therefore, needs to be measured across all programs. In mental health, some programs articulate PCC as a core aspect (23) and other more specified programs, such as assertive community treatment, have person-centered care as an explicit part of fidelity measurement (24). Given variety in the purpose, structure and intensity of mental health programs, the challenge is to find a shared practice across programs that reflects person-centered care. One such practice is service planning which produces a service plan, a form of documentation, that all programs utilize to map the course of care for service users. Evaluating service plans, while not a direct measure of the person-centered process, can provide a common indicator.

In mental health settings, the shift toward person-centered care has been driven by the recovery movement. Emerging in the 1980's from the voices of people with lived experience of mental illnesses, the recovery movement has challenged the prevailing paradigm of authoritative and paternalistic approaches to mental health care (25). More recently, recovery has shaped system transformation efforts after being endorsed by the U.S. policymakers. Person-centeredness is one of the fundamental components of recovery, which calls for care that acknowledges the unique recovery journey of each individual and is self-determined (26).

One recovery-oriented practice that has operationalized the delivery of person-centered care within mental health settings is Person-Centered Care Planning (PCCP) (27). This manualized intervention is anchored in service planning, which maps out a person's care and shapes his or her care experience. The aim of the planning process is to develop and implement an action plan to assist the person in achieving his or her unique, personal goals on the recovery journey. PCCP combines both the values of recovery and a well-specified collaborative approach to service planning. PCCP has explicitly been identified as a requirement by key funders of community mental health services (28) and a core standard of certified community behavioral health clinics established by the Excellence in Mental Health Act (29).

Recent efforts have identified five primary competency domains which support a fully person-centered planning process: 1) strengths-based, culturally informed, whole person-focused; 2) cultivating connections inside the system and out; 3) rights, choice, and control; 4) partnership, teamwork, communication, and facilitation; and 5) documentation, implementation, and monitoring (28). While the person-centered plan itself is directly related to the documentation domain, it is also an overall indicator and reflection of the other competency domains. A person-centered plan is rooted in a person-centered process which includes the therapeutic encounter and decision making.

When implementing PCCP at the provider level, the first step is to elicit and empathize with an individual's subjective experiences as a whole person and help them identify and articulate their interests, preferences, and personal recovery goals. Providers then translate conversations into the documentation of the person-centered plan itself. This includes reframing symptoms and impairments as barriers to goal attainment; reframing the use of medications as tools for overcoming these barriers and moving ahead in one‘s life; instilling hope and encouraging the person‘s incremental efforts in the face of fear, uncertainly, and demoralization; identifying short-term, realistic, and measurable objectives that can be achieved within the plan period of 3 to 6 months, while keeping these objectives explicitly connected to longer term aspirations that might span years; and expanding the action network to include natural supporters as well as professional providers. Providers address requirements for “medical necessity” criteria by offering methods of documentation that simultaneously honor what is most important to the individual while still incorporating elements from a health and safety perspective (27).

This study presents the development and validation of the Person-Centered Care Planning Assessment Measure, an objective measure of person-centered care that can be utilized as a clinical tool for quality improvement purposes. Based upon a randomized controlled trial of PCCP, the study tests the reliability and validity of the measure using a sample of service plans from community mental health clinics.

## Materials and Methods

The first phase of the study was the development of the scale and the second phase was psychometric testing of the scale. The parent study (ClinicalTrials.gov ID: NCT02299492) was approved by the New York University Institutional Review Board and was conducted 2013–2018.

### Development of the Measure

The PCCP-AM was created by the practice developers as a competency-based measure to evaluate the extent to which practitioners incorporate person-centered content within their required service plan documentation. It is organized around the following key plan components: goals, strengths and barriers, short-term objectives, supports, professional/ billable services, and natural support and self-directed actions. Each item is scored according to a four-point Likert scale: One (1) equals “needs improvement”; two (2) equals “approaches standard”; three (3) equals “meets standard”; four (4) equals “exceeds standard.” An initial 13-item measure of PCCP was developed based on a review of the literature including a white paper on person-centered planning commissioned by the Substance Abuse Mental Health Services Administration (30). The developers generated items that captured the most common domains of person-centered practice identified in the literature and informed by reviews of recovery plans and documentation requirements from over 25 states (31, 32). The initial draft measure was piloted in trainings and consultation efforts throughout the United States, including an initiative with the Texas Department of Mental Health to develop a standard recovery plan auditing tool for statewide quality monitoring efforts (33). Within this partnership, the measure was reviewed by a wide range of stakeholders including clinical practitioners, agency administrators, state office quality monitoring representatives and people with lived experience. This stakeholder review process was then followed by a 2-day, on-site auditing pilot where the draft PCCP-AM was applied to recovery plans with a diverse team of stakeholders carrying out side-by-side ratings, which led to further refinement of the items.

In the interests of parsimony and to develop a measure that would be feasible as a clinical tool, the PCCP-AM was further reduced to a 10-item measure by discarding three items. Specifically, an item on strengths in the assessment was discarded as it was deemed to be duplicative of another question evaluating the integration of strengths throughout the plan. A second item on Specific Measurable Attainable Relevant and Time-based criteria for short term objectives was discarded as it was duplicative of another item, which evaluates the specificity of the plan. Finally, a third item on person-first language was discarded as it was found to be less sensitive to variation than another similar item which asks about evidence of person's input into the plan. The final version of the PCCP-AM was a 10-item measure (see Table 1).

**Table 1:** Comparison of PCCP-AM items and expert rating instrument items.

**PCCP-AM item(s)**	**Expert rater item(s)**
(1) Presenting problem/barriers	Barriers and functional impairment clearly stated
(2) Narrative/interpretive summary	Cultural factors Stage of change Hypothesis Medical necessity
(3) Direct service user input	Client and family driven
(4) Goal statements	Goal statements
(5) Actively incorporates strengths	Strengths actively used
(6) Objectives go beyond service participation	Objectives linked to goals and barriers
(7) Target dates on short-term objectives	No corresponding item
(8) Natural supports and community engagement	Natural supports identified
(9) Interventions—who, what, when, why?	Interventions
(10) Self-directed action steps	Self-directed and Natural Support Action Steps

Collectively, these items capture the main domains of person-centered documentation which discriminate traditional service planning from recovery-oriented person-centered planning including: utilization of strengths throughout the plan; presenting problems as barriers to personal goals; having goal statements that focus on having a meaningful life; demonstrating direct input from the person; integrating cultural factors; ensuring community integration and use of informal supports; and specifying measurable individualized action steps by both provider and the person. These domains of person-centered documentation are consistent with practitioner core competency areas in person-centered planning (31) as well as federal regulations and guidelines which outline requirements for person-centered care in community mental health (28, 29).

### Psychometric Field Testing

Psychometric testing was conducted as part of a multi-site randomized controlled trial of PCCP (34). Reliability was tested using data collected from a chart review and validity was tested using data collected during the technical assistance phase of the PCCP training.

The parent study was set within community mental health clinics with seven sites randomized to the PCCP condition and seven to the control condition. These clinics were from two states with ~8,000 service users and provided a range of services including outpatient therapy, crisis intervention, medication management, case management, residential programs, community support programs, and rehabilitation services. Site eligibility criteria included serving people diagnosed with severe mental illnesses and no prior PCCP training. The provider sample consisted of 60 provider teams who retained the same supervisor throughout the study (out of a possible 81 teams trained in PCCP). Teams included one supervisor and two direct care staff nominated by the supervisor for their leadership capacity defined as being a role model and having potential to be a supervisor. The experimental sites received a 2-day in-person training session followed by monthly technical assistance over a 12-month period.

#### Internal Consistency

To evaluate the effectiveness of the training, chart reviews of service plans were conducted at experimental and control sites by researchers not blind to the intervention. Agency medical records staff, who were not members of the research team, were instructed to randomly select service plans from a list of study participants. Each service plan selected was from a unique service user. Based on power calculations for the RCT, 20 plans were selected from each of the 14 sites at three time points: 1 month prior to intervention baseline, 1 month prior to 12 months, and 1 month prior to 18 months. Due to low service user enrollment at one site, only 18 service plans (six from each timepoint) were selected. In total, 798 charts were randomly sampled. Three raters, two at each site, assessed the 798 service plans using the PCCP-AM. Two of the raters had master's level social work degrees and one had a bachelor's level degree. The raters were trained by PCCP experts on using the measure. They developed coding rules to guide scoring and met regularly to review their coding process.

Internal consistency was calculated using Cronbach's alpha for each of the three collection time points (*N* = 266), and for all three combined (*N* = 798). Cronbach's alpha was used as a conservative statistic for determining internal consistency as it calculated the lower bound of the internal consistency of the PCCP-AM (35).

#### Interrater Reliability

Interrater reliability was established by comparing PCCP-AM scores of a subsample of the 798 service plans. At each of the 14 clinic sites, 12 service plans were randomly selected to be coded by two raters yielding a total of 168 service plans, sufficient to test reliability while also being feasible for the raters.

Kendall's *W* was used to assess concordance among raters while correcting for ties, due to the non-parametric, ordinal nature of the data (36). In addition, agreement between raters of the 168 service plans was assessed by determining the percent of agreement between raters that was within ± one point. This analytic method was utilized because the PCCP-AM is designed for use as a quality improvement measure in routine care. The tool is designed for providers with different disciplines, education levels, licensing levels, and clinical or administrative experience and different types of agencies. Recognizing the possible wide variation in agency context and rater characteristics, we chose to determine the percent of scores ± one point in our analysis to better reflect interrater agreement in agency practice.

#### Concurrent Validity

Concurrent validity was established by comparing the assessment summaries of PCCP expert raters on service plans with the PCCP-AM scores. The sample size was determined by the technical assistance phase of the RCT study. Provider teams in the experimental condition submitted de-identified service plans for feedback during monthly technical assistance calls. A team from each of the seven experimental sites provided one service plan for each monthly call over a year, yielding a total of 84 care plans. Expert raters provided feedback to providers using a 14-item assessment instrument which included narrative feedback and a quantitative rating. Seven of these plans were excluded from the analysis as the primary diagnosis was not a mental health disorder and nine were excluded as the expert raters did not provide a numeric rating, resulting in a total sample of 68 service plans. Raters from the research team also rated these 68 service plans utilizing the PCCP-AM.

For construct validity, we hypothesized that the ratings of the PCCP-AM would be in concordance with expert ratings of the same service plans. To conduct a comparison between the expert rater instrument and the PCCP-AM, the 14 plan components of the expert rater instrument were mapped to the 10-item PCCP-AM (see Table 1). Two of the PCCP-AM items did not correspond one-to-one with expert rater instrument items. Four categories in the expert rating instrument corresponded to the narrative/interpretive summary in the PCCP-AM: cultural factors, stage of change, hypothesis/clinical interpretation, and medical necessity. The mean of these four ratings was calculated with equal weights. PCCP-AM Item 7, target date on short term objectives was not mapped onto any expert rater instrument item. PCCP-AM Scores were compared with expert rater scores by calculating the percent agreement within one point (±) for each item, in addition to using Kendall's *W* to assess concordance (36). The final sample resulted in 68 plans compared between expert consultants and PCCP-AM. Individual items had a range of 61 to 68 due to missing data, which was managed with pairwise deletion.

## Results

### Reliability

The overall internal consistency as measured by Cronbach's Alpha was α = 0.72 for 798 service plans. For 266 service plans collected at baseline, the internal consistency was α = 0.64. For 266 service plans collected at 12-months, the internal consistency was α = 0.74 and for 266 service plans collected at 18-months, the internal consistency was α = 0.73.

Interrater Reliability for each of the 10 PCCP-AM items as measured by coefficients of concordance ranged from *W* = 0.77 to *W* = 0.89, with four items being > 0.80 (see Table 2). The percent of scores within ± 1 point of each other ranged from 85.7 to 100% (see Table 2). All items with the exception of item 6 had 94% agreement or above. Item 7 had 100% agreement and items 2 and 4 had 98.8% agreement. Compared to the other nine items, Item 6 had much lower agreement between raters, with only 86.7% of scores being within ± 1 point of each other. Item 6 was also the only item to have <50% of scores in perfect agreement between raters. Items 1 and 8 had the second lowest percentage of scores within ± 1 point, at 94.0%. The mean percentage of scores between ± 1 was 95.5% and the median percentage was 95.8%. Only one item, Item 6 was more than two standard deviations from the mean, being 2.4 standard deviations less than the mean. Item 7 was the only item greater than one standard deviation above the mean. Neither Items 2 nor 7 had any differences in scores of ± 3, while Items 6, 8, and 9 each had differences of scores of both +3 and −3. Item 9 had the highest percentage of scores in perfect agreement, with 43.9% of scores having no difference between raters.

**Table 2:** Percentage differences and coefficients of concordance between two raters on PCCP-AM items.

	**% difference of ≤1**	**Mean of differences (SD)**	Coefficient of concordance^*^
Item 1	94.0	0.05 (0.69)	0.84
Item 2	98.8	0.07 (0.50)	0.89
Item 3	95.2	0.18 (0.73)	0.78
Item 4	98.8	−0.02 (0.64)	0.81
Item 5	96.4	0.11 (0.62)	0.81
Item 6	85.7	0.07 (0.86)	0.77
Item 7	100.0	0.12 (0.45)	0.85
Item 8	94.0	0.20 (0.76)	0.79
Item 9	97.6	0.01 (0.66)	0.83
Item 10	94.6	0.09 (0.76)	0.78

* *as calculated by Kendall's W*.

### Validity

Coefficients of concordance ranged from *W* = 0.55 to *W* = 0.74. One item fell below *W* = 0.60 and two were above *W* = 0.70 (see Table 3). The percent of PCCP-AM item scores within ± 1 point of expert rater scores ranged from 73.8% (Item 3) to 86.8% (Item 2), with all scores except for Item 3 being equal to or higher than 80% (see Table 3). For seven items, the raters more often scored lower than the experts and for two items the raters more often scored higher than the experts. Item 2 was 1.02 standard deviations above the mean, while Item 3 was 2.21 standard deviations below the mean. All other percentages were within 1 standard deviation of the mean percentage by item. Item 2 had the strongest relationship between scores on the PCCP-AM and expert rater scores, despite measuring multiple aspects of assessments. Item 9 was the only item to have more than 40% perfect agreement with a difference in scores of 0. All items had at least 20% perfect agreement. Item 2 had over 60% of scores with a difference of 1 between the PCCP-AM and expert raters and no scores where expert raters scored the charts 2 or 3 points higher than PCCP-AM raters. Item 8 had 40% of scores with a difference of −1 between PCCP-AM and expert raters, with no differences being +2 or +3 between the two. Only two items, Item 3 and Item 8 had any difference of −3 between PCCP-AM and expert raters, while seven items had some number of score differences of +3. Item 10 was the only item for all PCCP-AM and expert rater scores to reside within ± 2 of each other.

**Table 3:** Percentage differences and coefficients of concordance between PCCP-AM items and expert rating instrument items.

	**% difference of ≤1**	**% < −1**	**% > 1**	**Mean of differences (SD)**	Coefficent of concordance^*^
Item 1	80.9	1.5	17.6	0.47 (1.15)	0.60
Item 2	86.8	0.0	13.2	0.94 (.70)	0.69
Item 3	72.1	19.7	6.6	−0.46 (1.21)	0.55
Item 4	85.3	1.5	13.2	0.49 (1.00)	0.74
Item 5	85.1	7.5	7.5	0.13 (1.17)	0.61
Item 6	83.8	7.4	7.4	0.43 (1.12)	0.63
Item 7	na	na	na	na	na
Item 8	81.8	18.2	0.0	−0.68 (1.07)	0.61
Item 9	84.8	4.5	10.6	0.12 (1.05)	0.65
Item 10	80.3	3.0	16.7	0.39 (1.07)	0.74

* *as calculated by Kendall's W*.

*na, not applicable*.

## Discussion

The PCCP-AM demonstrated acceptable reliability and validity as a clinical tool to measure person-centered care. The measure, which was developed by PCCP experts who had authored the PCCP intervention, uses the service plan as an indicator of multiple dimensions of PCCP. When compared to the gold standard ratings by PCCP experts, the tool performed well overall with all but one item showing good concordance and falling within one point of the expert rating for more than 80% of the scores. The items 2 and 5 had the highest levels of validity showing that the measure was strongest in capturing competency in developing a plan that is strengths-based, culturally informed and whole person-focused. Item 9 also performed well, which measured competency in creating a plan that specifies the details of the intervention, in terms of what is done and by whom. These competencies are both key indicators of person-centered care and part of creating a plan that functions as a meaningful tool of accountability. These plans not only meet requirements for reimbursable services but also map out how an individual and their team work to support recovery. The weakest item in terms of validity was the item capturing direct service user input which may be the difficulty of operationalizing exactly how that is indicated in a service plan, whether it is inferred or should be stated explicitly.

The measure showed acceptable reliability, with an overall internal consistency of 0.72 across all service plans. Results revealed there was lower reliability in the sample of baseline service plans, perhaps indicating that the measure is more consistent when providers have been trained in the PCCP intervention. In terms of interrater reliability, all but one of the items was reliable within one point for 94% of the item scores. Raters disagreed to a greater extent about the item pertaining to whether the objectives go beyond service participation. There may have been ambiguity for the raters in terms of what constitutes activities beyond service participation or in determining the extent to which the objectives go beyond service participation.

By assessing person-centered care as indicated by the service plan, the PCCP-AM meets the need for an objective measure of PCC. While PCC encompasses the whole process of care inclusive of but not limited to documentation, the plan covers each of the core competency domains (e.g., strengths-based, culturally informed, whole person-focused, cultivating connections inside the system and out; rights, choice, and control; partnership, teamwork, communication, and facilitation; and documentation, implementation, and monitoring). While the plan itself is directly tied to the documentation domain, it is a strong indicator of other domains. However, measures of PCC should not be limited to a review of the service plan, as it is still possible that a plan can indicate a high level of person-centered care “on paper” but care in actuality could still be pathologizing and professionally driven. This need to capture process directly in quality measures still prevails, particularly through observational measures and integrating service user perspectives.

As a clinical tool, the PCCP-AM can be an important implementation strategy by facilitating ongoing monitoring and feedback to providers on their person-centered care practice. A recent synthesis of implementation frameworks lists ongoing implementation support strategies as: technical assistance, supervision and coaching and supportive feedback mechanisms (37). Similarly, Powell et al. (38) include developing and implementing tools for quality monitoring as a key implementation strategy. The PCCP-AM is an accessible tool for supervisors, coaches and technical assistance providers to monitor the delivery of person-centered care and provide feedback to clinicians. The measure can also be embedded in the electronic health record for documentation and quality improvement purposes by aggregating PCCP-AM scores across providers and programs.

There are several limitations to this study. The reduction of items in the interests of parsimony and feasibility may have restricted the breadth of the instrument. The psychometric analysis of the PCCP-AM focused on establishing validity for clinical utility and therefore, neither the interrater reliability nor concurrent validity were established using the most robust tests. This trade off had the positive effect of establishing how the PCCP-AM may be useable by clinicians and administrators with a variety of education levels and practice experience. The measure should undergo more robust psychometric testing to establish it as a research instrument. Lastly, the study is limited in its use of a single comparison measure for establishing validity which relied on expert rating. Future refinement should consider more sources of data to establish both concurrent and predictive validity.

### Conclusion

Based on a large study across multiple agencies, the PCCP-AM proved to be a reliable and valid measure of person-centered care as indicated by the service plan. The strength of the measure is that it is objective and can be applied across programs, making it a valuable tool to meet the increasing demands for documentation of person-centered care (39). The tool also can be utilized for clinical purposes by supervisors and coaches to monitor care and provide ongoing feedback. The PCCP-AM should be refined more to strengthen its validity and reliability by comparing it to independent assessments of care processes and by improving the calibration of the response set. In the meantime, the PCCP-AM provides an important step toward developing a clinically useful measure that captures person-centered care, a vital but often elusive aspect of service quality.

## Data Availability Statement

The raw data supporting the conclusions of this article will be made available by the authors, without undue reservation.

## Ethics Statement

The studies involving human participants were reviewed and approved by New York University Institutional Review Board. The participants provided their written informed consent to participate in this study.

## Author Contributions

VS designed the study and drafted the manuscript. DB, JT, LJ, AR, and SM drafted the manuscript. DB and SM developed the analytic strategy and conducted the data analyses. All authors reviewed and approved the manuscript.

## Conflict of Interest

SM has received consulting fees from Allergan and Sage Therapeutics. The remaining authors declare that the research was conducted in the absence of any commercial or financial relationships that could be construed as a potential conflict of interest.

## Publisher's Note

All claims expressed in this article are solely those of the authors and do not necessarily represent those of their affiliated organizations, or those of the publisher, the editors and the reviewers. Any product that may be evaluated in this article, or claim that may be made by its manufacturer, is not guaranteed or endorsed by the publisher.
