# Supplementary material for: Developing a Tool to Measure Person-Centered Care in Service Planning
Source: Front Psychiatry. 2021 Aug 2;12:681597. doi: 10.3389/fpsyt.2021.681597 (PMC8365091; doi:10.3389/fpsyt.2021.681597)
Supplement: Supplementary file 1 [file Data_Sheet_1.PDF]

Person-Centered Recovery Planning Quality Indicators

This quality indicators checklist will be used to evaluate the presence/absence of key PCRP documentation indicators.

One (1) equals "needs improvement"; two (2) equals "approaching standard"; three (3) equals "meets standard"; four (4) equals "exceed standard".

**Item #**	**Documentation Indicator**	**Scoring Instructions**	**1**	**2**	**3**	**4**
1	The assessment includes **a description of the presenting problem/barriers** as a result of the mental health or substance abuse issues to goal attainment. These are descriptions of functional impairments and symptoms as a result of the diagnosis	4 = There is a clear description of mental health and/or addictions-related barriers and how they interfere with life recovery goals, i.e., functional impairments are described.<br>3 = Mental health and substance use barriers are listed, but not in terms of functional impairments.<br>2 = Barriers are identified by listing diagnosis(es) only.<br>1 = There are no barriers, or the barriers identified are not related to mental health or addiction issues. They are only practical or resource barriers, e.g., lacks transportation, money, etc.				

©Janis Tondora, Psy.D (janis.tondora@yale.edu) and Diane Grieder, M.Ed (diane@alipar.org) 2011. Please contact primary authors for permission to use.

2	The **narrative/interpretive summary** includes the following required elements:<br>1. Strengths, interests, and current and/or desired life roles and priorities.<br>2. Any interfering perpetuating factors, e.g., trauma history, co-occurring medical or substance use disorders, etc. These are the <u>barriers</u> that get in the way of the person achieving their goal on their own and support medical necessity.<br>3. Individual's stage of change/stage of recovery (Stage of readiness for any relevant behavior change that could help them move towards their goal)<br>4. Available natural supports or community resources<br>5. Cultural factors and any impact on treatment<br><br>A clinical hypothesis/understanding/core theme re: what drives the individual's experience of illness and recovery -the "why" question	4= The summary includes <u>all</u> the required elements and is particularly well documented, painting a rich and individualized picture of the person's story.<br>3 = The summary is well documented but is missing one or two of the required elements.<br>2 = The summary is well documented but is missing 3 or more of the elements.<br>1 = There is no integrated summary present in the record.<br><br>If required elements are missing, please provide comments.				

©Janis Tondora, Psy.D (janis.tondora@yale.edu) and Diane Grieder, M.Ed (diane@alipar.org) 2011. Please contact primary authors for permission to use.

3	6. The plan/plan update is developed collaboratively and there is **evidence of direct input from the person**, e.g., the includes quotes from the individual and/or statements such as "Jose stated…" and there is evidence they were offered a copy of the plan (Note: This may be found in a progress note following the planning meeting or directly on the plan itself.)	4= The plan appears "person-driven". Excellent incorporation of the person's input (e.g., preferred goals, quotes, copy) throughout the plan.<br>3= There is some evidence of the person's input in the plan (i.e.., the goal(s) appear consistent with stated preferences, but may not include quotes or evidence of copy given).<br>2= The plan appears professionally driven even though there's a signature or evidence that a copy was given.<br>1= The plan appears professionally driven, no signature is included, no evidence that a copy was given.				
4	The **goal statements** on the plan/plan update are about having a meaningful life in the community, not only symptom reduction or compliance. Ideally, the goal reflects something "higher" – a valued community/life role that they want to obtain and are in the individual's own words. Ideally goals are reflected by "I" statements in quotes. "I'd like to join a choir." "I want a better relationship with my dad."	4= The goals are very life role oriented, beyond the management of symptoms/problems, and are in quotes (e.g., "I want to go back to work").<br>3 = The goals are life-oriented, but are not stated in the person's own words (e.g., Wanda will pursue employment).<br>2= The goals reflect the narrow management of clinical symptoms, problems, or use of treatment (e.g., Wanda will be less depressed, or Wanda will take her meds as prescribed, or "I know I need to be med compliant") or the goals are too vague (e.g., "I just want to be happy, normal").<br>1 = There are no goal statements on the plan or the goal statements are not life role oriented (i.e., they narrowly target problems/symptoms).				

5	The plan/plan update **actively incorporates the person's identified strengths** into the goals, objectives, or interventions/action steps.	4= Strengths are used in 2 or more places within the plan.<br>3 = Strengths are identified and used in 1 place within the plan.<br>2 = Strengths are identified in the plan but are not actively used anywhere in the goals, objectives, or action steps.<br>1 = Strengths do not appear to be used in the plan at all.				
6	Do the **objectives go beyond service participation**? i.e., Are they only about "will attend X,Y,Z services" or do they capture a positive/meaningful change in behavior/change in functioning/change in status? e.g., instead of framing the objective as "Client will regularly attend Dialectical Behavior Therapy" focus on the desired behavior change associated with that treatment intervention, e.g., "Jane will use Mindfulness skills to improve regulation of emotions as evidenced by having no more than 2 incidents of self-injurious cutting per week for the next 30 days."	4= All of the objectives go beyond service participation.<br>3= Most of the objectives go beyond service participation.<br>2 = Some of the objectives go beyond service participation.<br>1= None of the objectives go beyond service participation.				
7	The **target dates of short-term objectives** on the plan/plan update are individualized rather than all objectives defaulting to a standard update cycle, e.g., every 90 days. Not every objective should take exactly the same time to achieve. Ideal to have variation.	4= Dates on the objectives are all individualized per task and understanding of the individual.<br>3= Some dates on some objectives are individualized.<br>2= Dates exactly the same on all the objectives.<br>1= There are no dates at all on the objectives.				

©Janis Tondora, Psy.D (janis.tondora@yale.edu) and Diane Grieder, M.Ed (diane@alipar.org) 2011. Please contact primary authors for permission to use.

8	The plan/plan update describes attempts to help the person to **connect with chosen activities in the broader community** rather than relying on social supports coming solely from behavioral health agencies. (This item aims to reduce the amount of time people spend in segregated setting designed solely for people with mental illnesses)	4 = Excellent connection to natural community activities and relationships in the plan.<br>3= Some evidence of natural community activities and relationships in the plan.<br>2 = Limited evidence of natural community activities or relationships in the plan.<br>1 = No natural community activities or relationships are referenced in the plan.				
9	**Interventions** meet the criteria of the 4 W's: **who** (responsible professional), **what** (billable service), **when** (frequency, intensity & duration of service), and **why** (purpose and intent).	4 = All of the interventions meet most of the 4W criteria.<br>3 = Most of the interventions meet most of the 5W criteria.<br>2 = Some of the interventions meet most of the 5W criteria.<br>1= None of the interventions meet most of the 5W criteria.				

©Janis Tondora, Psy.D (janis.tondora@yale.edu) and Diane Grieder, M.Ed (diane@alipar.org) 2011. Please contact primary authors for permission to use.

10	The plan/plan update notes at least **one self-directed action step and at least one action step by natural supporters** (if preferred by the individual), as available. (Note: These are typically identified within the assessment process and build upon the person's strengths.)	Self-directed action steps are steps individuals can take themselves to help them accomplish their objectives. The person's Action Step should not merely be a "flipping" of the intervention statement as this does not add anything substantive to the plan. Instead, try to focus on the specific "value-added" task the person can pursue on his/her own. For example, NOT Client will consistently attend Creative Writing Rehab Group but instead Ingrid will journal for 15 minutes per day as this is an important wellness activity identified in her WRAP plan. Natural supports may be incorporated within the interventions/services sections of Recovery Plans but may also be evident in separate fields dedicated specifically to this purpose. Note that this item would be rated as N/A if it is the individual's stated preference NOT to include any natural supporters and this preference is stated in the Recovery Plan.<br><br>4=The plan includes and at least one natural supporter AND a self-directed action step (or have indicated prefer not to have them involved).<br>3= The plan includes a natural supporter action step OR a self-directed action step (or have indicated prefer not to have them involved).<br>2= The plan includes a self-directed action step OR a natural support, but they are poor in quality (i.e., vague or treatment focused).<br>1= No self-directed or natural supporter intervention steps are documented in the plan.				

©Janis Tondora, Psy.D (janis.tondora@yale.edu) and Diane Grieder, M.Ed (diane@alipar.org) 2011. Please contact primary authors for permission to use.
